# Comparison of Childbirth Delivery Outcomes and Costs of Care Between Women Experiencing vs Not Experiencing Homelessness

**DOI:** 10.1001/jamanetworkopen.2021.7491

**Published:** 2021-04-22

**Authors:** Ayae Yamamoto, Lillian Gelberg, Jack Needleman, Gerald Kominski, Sitaram Vangala, Atsushi Miyawaki, Yusuke Tsugawa

**Affiliations:** 1 Department of Health Policy and Management, University of California, Los Angeles Fielding School of Public Health; 2 Department of Healthcare Economics, UnitedHealthcare, Cypress, California; 3 Department of Family Medicine, David Geffen School of Medicine at University of California, Los Angeles; 4 Office of Healthcare Transformation and Innovation, VA Greater Los Angeles Healthcare System, Los Angeles, California; 5 University of California, Los Angeles Center for Health Policy Research; 6 Division of General Internal Medicine and Health Services Research, David Geffen School of Medicine at University of California Los Angeles; 7 Department of Public Health, Graduate School of Medicine, University of Tokyo, Bunkyo-ku, Tokyo, Japan

## Abstract

**Importance:**

Women and families constitute the fastest-growing segments of the homeless population. However, there is limited evidence on whether women experiencing homelessness have poorer childbirth delivery outcomes and higher costs of care compared with women not experiencing homelessness.

**Objective:**

To compare childbirth delivery outcomes and costs of care between pregnant women experiencing homelessness vs those not experiencing homelessness.

**Design, Setting, and Participants:**

This cross-sectional study included 15 029 pregnant women experiencing homelessness and 308 242 pregnant women not experiencing homelessness who had a delivery hospitalization in 2014. The study used statewide databases that included all hospital admissions in 3 states (ie, Florida, Massachusetts, and New York). Delivery outcomes and delivery-associated costs were compared between pregnant women experiencing homelessness and those not experiencing homelessness cared for at the same hospital (analyzed using the overlap propensity-score weighting method and multivariable regression models with hospital fixed effects). The Benjamini-Hochberg false discovery rate procedure was used to account for multiple comparisons. Data were analyzed from January 2020 through May 2020.

**Exposure:**

Housing status at delivery hospitalization.

**Main Outcomes and Measures:**

Outcome variables included obstetric complications (ie, antepartum hemorrhage, placental abnormalities, premature rupture of the membranes, preterm labor, and postpartum hemorrhage), neonatal complications (ie, fetal distress, fetal growth restriction, and stillbirth), delivery method (ie, cesarean delivery), and delivery-associated costs.

**Results:**

Among 15 029 pregnant women experiencing homelessness (mean [SD] age, 28.5 [5.9] years) compared with 308 242 pregnant women not experiencing homelessness (mean [SD] age, 29.4 [5.8] years) within the same hospital, those experiencing homelessness were more likely to experience preterm labor (adjusted probability, 10.5% vs 6.7%; adjusted risk difference [aRD], 3.8%; 95% CI, 1.2%-6.5%; adjusted *P* = .03) and had higher delivery-associated costs (adjusted costs, $6306 vs $5888; aRD, $417; 95% CI, $156-$680; adjusted *P* = .02) compared with women not experiencing homelessness. Those experiencing homelessness also had a higher probability of placental abnormalities (adjusted probability, 4.0% vs 2.0%; aRD, 1.9%; 95% CI, 0.4%-3.5%; adjusted *P* = .053), although this difference was not statistically significant.

**Conclusions and Relevance:**

This study found that women experiencing homelessness, compared with those not experiencing homelessness, who had a delivery and were admitted to the same hospital were more likely to experience preterm labor and incurred higher delivery-associated costs. These findings suggest wide disparities in delivery-associated outcomes between women experiencing homelessness and those not experiencing homelessness in the US. The findings highlight the importance for health care professionals to actively screen pregnant women for homelessness during prenatal care visits and coordinate their care with community health programs and social housing programs to make sure their health care needs are met.

## Key Points

**Question:**

Do pregnant women experiencing homelessness have poorer childbirth delivery outcomes and higher costs of care compared with pregnant women not experiencing homelessness?

**Findings:**

In this multistate population-based cross-sectional study of 15 029 pregnant women experiencing homelessness and 308 242 pregnant women not experiencing homelessness, those experiencing homelessness were more likely to experience preterm labor and had higher delivery-associated costs. Those experiencing homelessness were also more likely to experience placental abnormalities, although this difference was not statistically significant.

**Meaning:**

These findings suggest that, within the same hospital, housing status is associated with preterm labor and higher delivery costs.

## Teaser

This cross-sectional study compares childbirth delivery outcomes and costs of care among women experiencing homelessness vs those not experiencing homelessness.

## Introduction

More than 560 000 individuals in the United States experience homeless on any given night,^1^ and each year, an estimated 1% of the US population experience homelessness.^2^ Homelessness increased in metropolitan cities in 2019 compared with 2018,^1^ and it has become a major concern that has received attention for funding for housing initiatives at the local and national levels.^3,4^ Individuals who are experiencing unstable housing or homelessness have higher disease burdens and mortality rates and 2.5-fold higher health care spending vs a comparable housed population.^5,6,7,8,9,10^

While most people experiencing homelessness are men, approximately 1 in 4 individuals experiencing homelessness are women or girls.^1^ More importantly, women and families constitute the fastest-growing segment of the homeless population.^11^ Circumstances associated with homelessness, such as malnutrition, and preexisting conditions, including substance abuse and mental illness, are associated with poorer health outcomes of the mother and fetus.^12,13^ Women experiencing homelessness are more likely to have dire living situations, which pose additional challenges that limit the ability of these women to take care of their health or seek care.^14^

There is limited evidence on whether pregnant women experiencing homelessness also experience poorer childbirth-associated outcomes and higher costs of care compared with women not experiencing homelessness. The current literature suggests that homelessness among pregnant women is associated with increased barriers to accessing prenatal care^15^ and fewer prenatal care visits.^16^ It is also associated with an increased likelihood of having infants who are born preterm, longer hospital lengths of stay, and increased likelihood of having babies who have low birth weight, are small for their gestational age, and need neonatal intensive care.^16,17,18^ However, existing research is limited to small studies conducted at a single hospital,^17,19^ city (ie, Los Angeles),^18^ or state (ie, Massachusetts).^20^ Therefore, whether their findings are generalizable to other regions of the US remains unclear. Moreover, prior studies compared women experiencing homelessness vs those not experiencing homelessness across hospitals; therefore, observed disparities in health outcomes may be associated with differences in the quality of hospitals from which women sought care.^20^ Thus, robust evidence on health outcomes and costs of care among these women using population-level data from multiple states is critically important. Given this context, using state-wide databases that include all hospital discharges from 3 large and diverse states, we examined the association between homelessness and obstetric, neonatal, and health service outcomes among all pregnant women who had a delivery hospitalization in 2014.

## Methods

The University of California-Los Angeles institutional review board deemed this cross-sectional study to not be human subject research and therefore exempt from institutional review board approval and informed consent. This study followed the Strengthening the Reporting of Observational Studies in Epidemiology (STROBE) reporting guideline.

### Data Sources and Study Sample

We analyzed the 2014 State Inpatient Database (SID) and the State Emergency Department Databases for 3 states (ie, Florida, Massachusetts, and New York) made available by the Healthcare Cost and Utilization Project of the Agency for Healthcare Research and Quality^21^ (see eAppendix in the Supplement for further discussion of these databases and excluded states). For each inpatient and hospital-affiliated emergency department discharge, there is an indicator for each patient's housing status reported directly from the hospitals, and this variable has been used in previous studies to identify the housing status of patients.^22,23,24,25,26^ Additionally, we used the 2016 American Hospital Association's Annual Survey Database^27^ and the 2014 Medicare Cost Reports^28^ for data on hospital characteristics.

The study population was restricted to women ages 18 to 44 years who had a hospitalization for delivery in 2014. The analysis was restricted to the first hospitalization. We excluded women who were missing housing status or any of the key adjustment variables described in a following section. Childbirth deliveries were identified using the enhanced method for identifying deliveries as described elsewhere.^29,30^ This method uses a combination of *International Classification of Diseases, Ninth Revision, Clinical Modification* (*ICD-9*) diagnosis and procedure codes and diagnosis-related group codes and has been found to be less likely to miss severe obstetric complications compared with a standard method of using V27 codes.^29^ See eTable 1 in the Supplement for a list of codes.

### Outcome Variables

Outcome variables were classified into 4 groups: (1) obstetric complications during pregnancy (ie, antepartum hemorrhage and placental abnormalities) or associated with labor (ie, premature rupture of membranes, preterm labor, and postpartum hemorrhage), (2) neonatal complications (ie, fetal distress, fetal growth restriction, and stillbirth), (3) delivery method (ie, cesarean delivery), and (4) delivery-associated costs. Delivery-associated costs were defined as the total cost of care of the delivery hospitalization. We used Healthcare Cost and Utilization Project’s cost-to-charge ratio file to calculate costs from total charges available in the SID. All costs were inflated to 2019 dollars using the gross domestic product price index. Childbirth delivery outcomes have been defined in prior studies.^31,32^ See eTable 2 in the Supplement for a list of codes.

### Adjustment Variables

In our multivariable regression models, we adjusted for patient-level adjustment variables and hospital fixed effects. Patient-level adjustment variables included age (categorized as 18-24 years, 25-29 years, 30-34 years, 35-39 years, and 40-44 years), race/ethnicity (ie, non-Hispanic White, non-Hispanic Black, Hispanic, and other race/ethnicity), primary insurance type (ie, Medicare, Medicaid, private, self-pay/no charge/other), smoking history (ie, ever smoked), multiple birth status, and 26 comorbidities included in the Elixhauser Comorbidities Index^33^ (similar medical conditions were combined while ulcer and AIDS were excluded from the index owing to low cell counts [ie, <3]). Smoking status was identified using *ICD-9* codes because these codes have been found to be reliable indicators associated with smoking status.^34^ In the cesarean delivery outcome model, we additionally adjusted for previous cesarean delivery. Hospital fixed effects (ie, indicator variables for each hospital) account for both measured and unmeasured (ie, time-invariant) characteristics of hospitals, allowing us to compare women experiencing and not experiencing homelessness within the same hospital.^35^

### Statistical Analysis

To control for sample selection bias, we used a doubly robust overlap propensity score–weighing method.^36,37^ In the propensity score and the outcome regression models, we included all patient-level adjustment variables explained in the previous section. The outcome regression models additionally adjusted for hospital fixed effects.

We used the overlap propensity score–weighting method, a new form of balancing weights using propensity scores, given that the distributions of propensity score showed limited overlap and extreme propensity scores. That is, there were small propensity scores for some individuals experiencing homelessness, which made it difficult to find the matched control in propensity score–matching method and led to inflated inverse weights when using inverse probability of treatment weights.^36,37,38^ The overlap weighting method upweights observations with the largest overlap in observed characteristics between 2 groups while downweighting those observations with the least overlap. In other words, it deemphasizes observations with propensity scores close to 0 or 1 and emphasizes those close to a propensity score of 0.5. An advantage of using this method over trimming methods is that it does not rely on arbitrarily selecting a cutoff point for the weights and does not involve removing observations. These weights provide estimates of the average treatment effect in the overlap population.^37^

Next, we used multivariable linear probability models with Huber-White heteroscedasticity-robust standard errors. We could not use logistic regression models because small cell sizes for some combinations of patient characteristics resulted in complete or quasicomplete separation.^39^ Delivery-associated costs were analyzed using generalized linear models with log-link and gamma distribution. We adjusted for patient-level variables (ie, age, race/ethnicity, primary payer, smoking history, multiple birth status, and Elixhauser comorbidities) and hospital fixed effects. After fitting the regression models, adjusted outcomes were calculated using the marginal standardization form of predictive margins (also known as predictive margins or margins of responses); for each individual, we calculated estimated probabilities of each outcome with housing status fixed at each category (ie, 0 or 1) and then found the mean over the distribution of covariates in our sample.^40^

We conducted several sensitivity analyses. First, because women experiencing homelessness are more likely to have limited and fragmented access to health care and thus may be less likely to have coded diagnoses of comorbidities, we conducted analyses accounting for these differences. To investigate how differential coding patterns between women experiencing and not experiencing homelessness may have been associated with changes to our findings, we reanalyzed the data without adjusting for Elixhauser comorbidities in our regression models. Second, given that women not experiencing homelessness may differ from those experiencing homelessness in many ways that cannot be accounted for by adjusting for observable characteristics, we used a comparison group consisting of women not experiencing homelessness who were in the lowest quartile of median household income by zip code (ie, who were low-income housed). Third, we examined whether outcomes differed between women experiencing and not experiencing homelessness when they were compared across hospitals using multivariable regression models without hospital fixed effects. Fourth, to determine how different modeling approaches may be associated with the magnitude and direction of delivery-associated costs, these costs were separately analyzed using negative binomial regression (ie, a log-link, negative binomial generalized linear model) and ordinary least squares with Huber-White robust heteroscedasticity standard errors.

All analyses were conducted in SAS Enterprise Guide statistical software version 4.2 (SAS Institute) and Stata statistical software version 14 (StataCorp). Because our study had multiple outcome variables, we accounted for multiple comparisons using the Benjamini-Hochberg procedure, setting the false discovery rate threshold to q < .05.^41^ This method controls the expected proportion of false positives to less than 5% and is less conservative compared with family-wise error rate controlling procedures, such as the Bonferroni procedure. Reported *P* values are adjusted and 2-sided. Data were analyzed from January 2020 through May 2020.

## Results

Our final sample consisted of 15 029 pregnant women experiencing homelessness and 308 242 pregnant women not experiencing homelessness. Women experiencing homelessness, compared with women not experiencing homelessness, were younger (mean [SD] age, 28.5 [5.9] years vs 29.4 [5.8] years); more likely to have Medicaid as the primary payer (14 695 women [97.8%] vs 145 264 women [47.1%]); and more likely to be non-Hispanic Black (4906 women [32.6%] vs 57 560 women [18.7%]), Hispanic (5495 women [36.6%] vs 57 315 women [18.6%]), or other race/ethnicity (3790 women [25.2%] vs 47 432 women [15.4%]). Women experiencing homelessness were also more likely to have coded diagnoses of hypertension, coagulopathy, obesity, alcohol abuse or liver disease, and psychoses. They were less likely to have ever been a smoker, have multiple births, and have coded diagnoses of hypothyroidism, deficiency anemias, or depression (Table 1). Women experiencing homelessness were more likely to be seen in small hospitals (ie, 1-99 beds) to medium-size hospitals (ie, 100-300 beds), hospitals with minor teaching status, government-owned hospitals, and hospitals most likely to be safety net hospitals (Table 2).

**Table 1.** Patient Characteristics by Housing Status (Descriptive)

Characteristic	Unweighted No. (%)		Weighted %	
	Experiencing homelessness (n = 15 029)	Not experiencing homelessness (n = 308 242)	Experiencing homelessness (n = 15 029)	Not experiencing homelessness (N = 308 242)
Age, mean (SD), y	28.5 (5.9)	29.4 (5.8)	28.4 (5.9)	28.4 (5.9)
Payer				
Medicare	83 (0.6)	1828 (0.6)	0.6	0.6
Medicaid	14 695 (97.8)	145 264 (47.1)	97.5	97.5
Private insurance	227 (1.5)	151 021 (49.0)	1.7	1.7
Self-pay, no charge, or other	24 (0.2)	10 129 (3.3)	0.2	0.2
Race/ethnicity				
Non-Hispanic White	838 (5.6)	145 935 (47.3)	6.2	6.2
Non-Hispanic Black	4906 (32.6)	57 560 (18.7)	32.7	32.7
Hispanic	5495 (36.6)	57 315 (18.6)	36.2	36.2
Other^a^	3790 (25.2)	47 432 (15.4)	24.8	24.8
Ever smoker	377 (2.5)	18 212 (5.9)	2.7	2.7
Multiple births	202 (1.3)	6678 (2.2)	1.4	1.4
Hypertension	423 (2.8)	7065 (2.3)	2.8	2.8
Neurological disorder	99 (0.7)	2148 (0.7)	0.7	0.7
Chronic pulmonary disease	750 (5.0)	15 024 (4.9)	5.1	5.1
Diabetes	206 (1.4)	3076 (1.0)	1.3	1.3
Hypothyroidism	253 (1.7)	10 963 (3.6)	1.7	1.7
Heart, circulation, or vascular disease	32 (0.2)	1231 (0.4)	0.2	0.2
Coagulopathy	550 (3.7)	8401 (2.7)	3.5	3.5
Obesity	1436 (9.6)	20 154 (6.5)	9.4	9.4
Fluid and electrolyte disorders	92 (0.6)	1647 (0.5)	0.6	0.6
Chronic blood loss anemia	1948 (13.0)	40 303 (13.1)	13.4	13.4
Deficiency anemia	1311 (8.7)	29 900 (9.7)	9.1	9.1
Alcohol abuse or liver disease	138 (0.9)	1137 (0.4)	0.8	0.8
Drug abuse	271 (1.8)	6395 (2.1)	1.9	1.9
Psychosis	246 (1.6)	2850 (0.9)	1.6	1.6
Depression	225 (1.5)	7058 (2.3)	1.5	1.5

^a^ Includes Asian or Pacific Islander individuals, Native American individuals, and individuals with other race/ethnicity.

**Table 2.** Hospital Characteristics by Housing Status (Descriptive)

Characteristic^a^	No. (%)	
	Experiencing homelessness (n = 15 029)	Not experiencing homelessness (n = 308 242)
Large hospital^b^	8400 (55.9)	219 275 (71.7)
Teaching status		
Major	323 (2.2)	147 331 (48.2)
Minor	14 615 (97.3)	104 560 (34.2)
Nonteaching	91 (0.6)	53 767 (17.6)
Control type		
Investor owned and for profit	82 (0.6)	36 750 (12.0)
Nongovernment owned and not for profit	452 (3.0)	235 552 (77.1)
Government owned and nonfederal	14 495 (96.5)	33 356 (10.9)
Safety net status^c^	14 733 (98.1)	128 153 (42.7)

^a^ These hospital characteristics were not included in the propensity score model or outcome model.

^b^ Large hospital was defined as 400 or more beds vs small hospital (ie, 1-99 beds) and medium-size hospital (ie, 100-399 beds).

^c^ A hospital was considered a safety net if it was in the highest quartile of Medicare disproportionate share hospital patient percentage for each state.

### Association Between Homelessness and Delivery Outcomes

**Figure 1.**
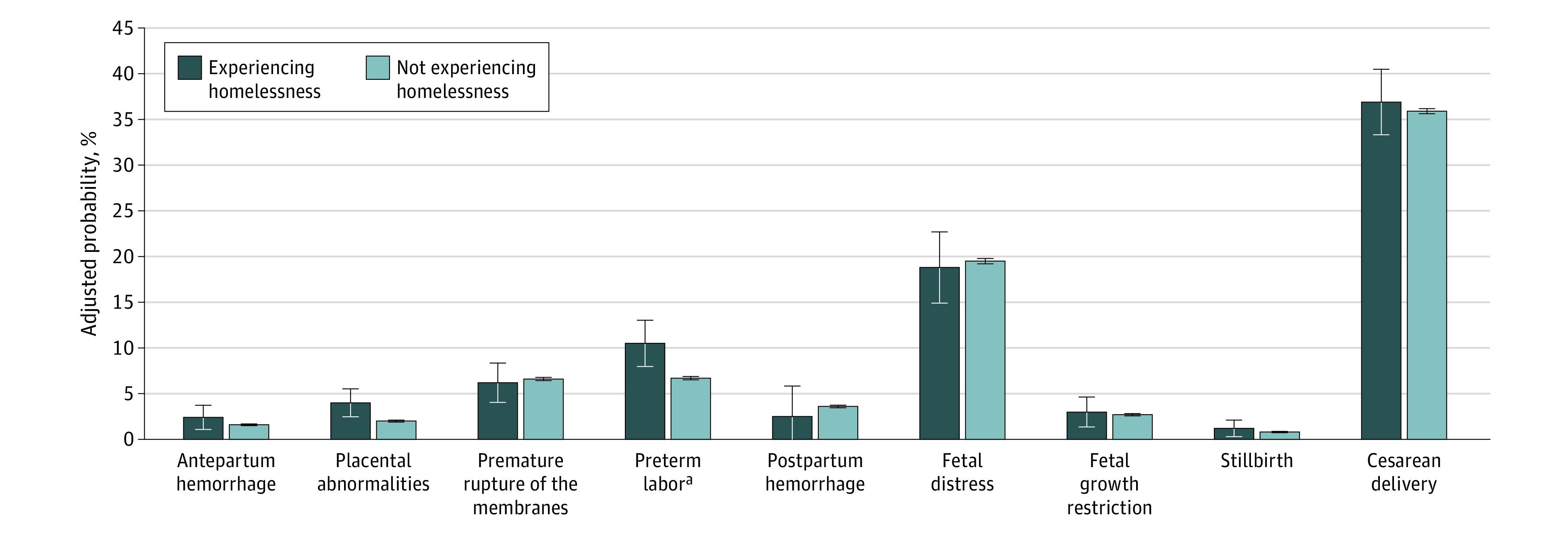
Patient Outcomes Adjusted for patient-level variables (ie, age, race/ethnicity, payer, smoking history, multiple birth status, and Elixhauser comorbidities) and hospital fixed effects. The cesarean delivery model was additionally adjusted for previous cesarean delivery. ^a^*P* value adjusted for multiple comparisons using Benjamini-Hochberg false discovery rate was statistically significant at 5%. Adjusted *P* = .03.

**Figure 2.**
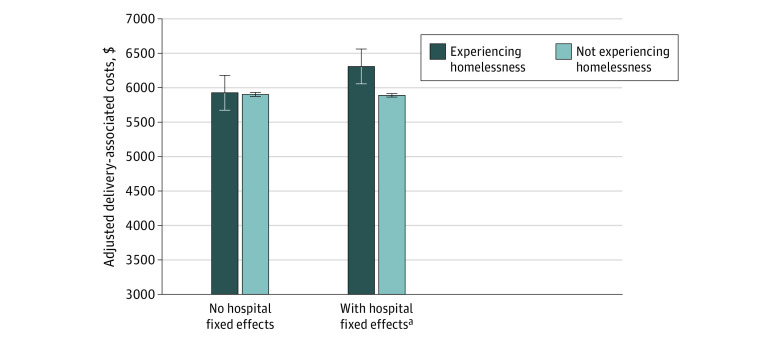
Delivery-Associated Hospitalization Costs Adjusted for patient-level variables (ie, age, race/ethnicity, payer, smoking history, multiple birth status, and Elixhauser comorbidities). Costs were inflated to 2019 dollars using the gross domestic product price index. ^a^*P* value adjusted for multiple comparisons using Benjamini-Hochberg false discovery rate was statistically significant at 5%. Adjusted *P* = .02.

After adjusting for patient characteristics and hospital fixed effects, we found that women experiencing homelessness, vs those not experiencing homelessness, had a higher likelihood of preterm labor (adjusted probability, 10.5% vs 6.7%; adjusted risk difference [aRD], 3.8%; 95% CI, 1.2%-6.5%; adjusted *P* = .03) and had higher delivery-associated costs (adjusted costs, $6306 vs $5888; aRD, $417; 95% CI; $156-$680; adjusted *P* = .02). Women experiencing homelessness also had a higher probability of placental abnormalities, although this difference was not statistically significant (adjusted probability, 4.0% vs 2.0%; aRD, 1.9%; 95% CI, 0.4%-3.5%; adjusted *P* = .053) (Figure 1 and Figure 2; eTable 3 in the Supplement).

### Sensitivity Analyses

Our findings were qualitatively unchanged by the analysis, excluding Elixhauser comorbidities, or by the analysis comparing women experiencing homelessness with women not experiencing homelessness who were low income (eTable 4 and eTable 5 in the Supplement). When we compared women experiencing homelessness with women not experiencing homelessness across hospitals (using weighted regression models without hospital fixed effects), we found that those experiencing homelessness had a lower likelihood of a cesarean delivery but a higher likelihood of fetal distress. We found no evidence that delivery-associated costs differed between the 2 groups comparing across hospitals (Figure 2; eTable 6 in the Supplement). We obtained a similar delivery-associated cost outcome with a negative binomial model and a greater cost difference with an ordinary least squares model (eTable 7 in the Supplement).

## Discussion

Using statewide data sets of all hospital admissions from 3 large and diverse states, this cross-sectional study found that pregnant women experiencing homelessness had a higher likelihood of experiencing preterm labor and higher delivery-associated costs compared with pregnant women not experiencing homelessness, even when they were cared for within the same hospital. Pregnant women experiencing homelessness also had a higher probability of placental abnormalities, although this difference was not statistically significant.

Given that we compared women experiencing homelessness with those not experiencing homelessness at the same hospital, observed differences in delivery outcomes and costs may be associated with disparities in the quality of care these 2 groups of women received at the same hospital, as well as unmeasured risk factors of women experiencing homelessness. For instance, the higher preterm labor rates we observed among women experiencing homelessness may be associated with other unmeasured clinical characteristics, such as infections, stressful life events, drug and alcohol use, history of preterm labor, and shorter time intervals between pregnancies, among others.^42^ Known risk factors for placental abnormalities include cocaine use, having a placental previa from a previous pregnancy, and previous uterine surgery, among others.^43^ Given that women experiencing homelessness are less likely to receive prenatal care^16^ and more likely to abuse alcohol and illicit drugs,^12,13^ it is possible that these women are presenting with heightened risk at the time of delivery.

Placental abnormalities (ie, previa, abruptio, and accreta) can lead to further complications, such as heavy bleeding, preterm birth, and stillbirth,^44,45^ and preterm labor can result in preterm births.^46^ Our findings were consistent with single-facility studies that found that mothers experiencing homelessness were more likely than those not experiencing homelessness to have adverse delivery outcomes, such as preterm delivery, an infant with low birth weight, and an infant who was small for gestational age.^17,19^ Such adverse delivery outcomes can be associated with detrimental outcomes for the mother and the infant over the long term.^47,48^

Our findings that women experiencing homelessness had worse delivery-associated outcomes suggest that these women need to be identified and be connected to resources to help springboard them out of homelessness. The Centers for Medicaid & Medicare Services established the Accountable Health Communities Model, which provides funding for selected clinic-community collaborations to address social determinants of health through efforts such as screening patients at clinics for social needs.^49^ However, less than a quarter of physicians screen patients for all 5 social needs: food insecurity, housing instability, unmet needs for utility, transportation needs, and interpersonal violence.^50^ Health care policies should encourage clinicians to screen pregnant women for social needs and collaborate with community resources, such as local housing authorities, so that pregnant women’s housing needs are met and they are able to receive care for their unmet health care needs.

There are several plausible explanations for why we did not observe significant differences for some of the outcomes we studied (ie, stillbirth, antepartum hemorrhage, fetal growth restriction, and postpartum hemorrhage). First, they are rarer events compared with preterm labor, and therefore, our study may be underpowered to detect significant differences in the rate of these outcomes. It is also possible that some outcomes are associated largely with the quality of care of the hospital where the women delivered (with small within-hospital variations), with no differences to be observed when we compared women experiencing homelessness vs not experiencing homelessness at the same hospital. This hypothesis is supported by our finding from the sensitivity analysis excluding hospital fixed effects of higher rates of fetal distress and lower rates of cesarean delivery for women experiencing homelessness. Furthermore, women experiencing homelessness had higher delivery-associated costs compared with women not experiencing homelessness within the same hospitals but not compared across hospitals. These findings suggest that women experiencing homelessness may have received delivery-associated care in hospitals that have lower mean delivery costs, higher fetal distress rates, and lower cesarean delivery rates, perhaps associated with these institutions’ limited resources and the high-risk populations they serve.

To our knowledge, this is one of few studies using multiple states to compare childbirth delivery outcomes and costs of care between women experiencing homelessness and those not experiencing homelessness. Prior studies^16,17,18^ have found that pregnant women experiencing homelessness were more likely to have adverse delivery outcomes, such as giving birth to infants who are born preterm, have low birth weight, or are small for gestational age. However, most existing studies^17,18,19,20^ have been limited in sample size and were conducted in a single state, city, or hospital. A population-level study^16^ using the Pregnancy Risk Assessment Monitoring System survey database from 31 states found that infants born to women experiencing homelessness had worse neonatal outcomes compared with infants born to women not experiencing homelessness. However, the study did not assess obstetric delivery complications or link survey data to hospital admission data. In our study, we included all hospital admission data from 3 states and compared obstetric, fetal, and health service outcomes. Our statistical models adjusted for hospital fixed effects, which allowed us to make robust within-hospital comparisons. Additionally, we used a doubly robust overlap propensity score–weighting method, which has the advantage of selecting a clinically relevant target population, achieving covariate balance between groups, and improving precision compared with other propensity score methods.^38^

### Limitations

This study has several limitations. First, as for any cross-sectional study, the temporality between exposure and outcome could not be assessed. However, women were identified as experiencing homelessness at the time of discharge in our data; therefore, it is unlikely that their delivery complications led them to become homeless. Second, with administrative data, exposure and outcome misclassifications are possible. For instance, the severity of homelessness may not be captured fully in our data. However, if women experiencing homelessness for a short time were coded as experiencing homelessness long term or if housing status was underreported, this would bias our estimates toward the null. In addition, concerns about the overconcentration of individuals experiencing homelessness in some hospitals were addressed by the inclusion of hospital fixed effects. Third, while we adjusted for a broad set of patient demographic and clinical variables in our analyses, we were unable to adjust for other potential confounders, such as maternal body mass index, history of preterm birth, and number of prenatal care visits.^51^ Fourth, while our study includes all women experiencing homelessness and not experiencing homelessness who had a delivery hospitalization from 3 states, our findings may not be generalizable to individuals experiencing homelessness in states not included in our analysis or individuals who were not hospitalized for deliveries. We were limited by the availability of the state data and homeless indicator data in states that were not included in our study. On the West Coast, individuals experiencing homelessness are able to live outside of shelters year round owing to milder climates, and therefore may not be able to access social services as readily as these individuals living in other states.^52^

## Conclusions

Using state-wide databases from 3 large states, we found that pregnant women experiencing homelessness were more likely to have preterm labor and had higher costs of delivery compared with pregnant women not experiencing homelessness who were cared for at the same hospital. These findings emphasize the importance of hospitals and clinicians to screen pregnant patients for social needs, including housing, and leverage community resources as soon as possible so that mothers can prioritize their health for safe delivery.

## Notes

Supplement. **eAppendix.** Database Details and States Excluded From Study **eTable 1.** *International Classification of Diseases, Ninth Revision* (*ICD-9*) Diagnosis and Procedure Codes and Diagnosis-Related Group (DRG) Codes Used to Define Delivery Hospitalizations **eTable 2.** *International Classification of Diseases, Ninth Revision* (*ICD-9*) Diagnosis, and Procedure Codes Used to Define Delivery Outcomes **eTable 3.** Patient Outcomes **eTable 4.** Patient Outcomes Excluding Comorbidities **eTable 5.** Patient Outcomes in Women Experiencing Homelessness vs Women Who Were Low Income and Housed **eTable 6.** Patient Outcomes Without Adjusting for Hospital Fixed Effects (Comparing Across Hospitals) **eTable 7.** Delivery-Associated Costs by Different Modeling Approaches
