# Supplementary material for: Comparison of Childbirth Delivery Outcomes and Costs of Care Between Women Experiencing vs Not Experiencing Homelessness
Source: JAMA Netw Open. 2021 Apr 22;4(4):e217491. doi: 10.1001/jamanetworkopen.2021.7491 (PMC8063065; doi:10.1001/jamanetworkopen.2021.7491)
Supplement: Supplement. — eAppendix. Database Details and States Excluded From Study eTable 1. International Classification of Diseases, Ninth Revision (ICD-9) Diagnosis and Procedure Codes and Diagnosis-Related Group (DRG) Codes Used to Define Delivery Hospitalizations eTable 2. International Classification of Diseases, Ninth Revision (ICD-9) Diagnosis, and Procedure Codes Used to Define Delivery Outcomes eTable 3. Patient Outcomes eTable 4. Patient Outcomes Excluding Comorbidities eTable 5. Patient Outcomes in Women Experiencing Homelessness vs Women Who Were Low Income and Housed eTable 6. Patient Outcomes Without Adjusting for Hospital Fixed Effects (Comparing Across Hospitals) eTable 7. Delivery-Associated Costs by Different Modeling Approaches [file jamanetwopen-e217491-s001.pdf]

Supplemental Online Content

Yamamoto A, Gelberg L, Needleman J, et al. Comparison of childbirth delivery outcomes and costs of care between women experiencing vs not experiencing homelessness. *JAMA Netw Open*. 2021;4(4):e217491. doi:10.1001/jamanetworkopen.2021.7491

eAppendix. Database Details and States Excluded From Study
eTable 1. *International Classification of Diseases, Ninth Revision* (*ICD-9*) Diagnosis and Procedure Codes and Diagnosis-Related Group (DRG) Codes Used to Define Delivery Hospitalizations
eTable 2. *International Classification of Diseases, Ninth Revision* (*ICD-9*) Diagnosis, and Procedure Codes Used to Define Delivery Outcomes
eTable 3. Patient Outcomes
eTable 4. Patient Outcomes Excluding Comorbidities
eTable 5. Patient Outcomes in Women Experiencing Homelessness vs Women Who Were Low Income and Housed
eTable 6. Patient Outcomes Without Adjusting for Hospital Fixed Effects (Comparing Across Hospitals)
eTable 7. Delivery-Associated Costs by Different Modeling Approaches

This supplemental material has been provided by the authors to give readers additional information about their work.

© 2021 Yamamoto A et al. *JAMA Network Open.*

eAppendix. Database Details and States Excluded From Study

The State Inpatient Database (SID) includes all inpatient discharge records from community hospitals (including emergency visits that resulted in hospitalization), and the State Emergency Department Database (SEDD) includes all emergency department visits at hospital-affiliated emergency departments that do not lead to a subsequent hospitalization. These databases capture visit information for all patients regardless of the type of insurance and insurance status. Records for each patient include key demographic information such as age, gender, race/ethnicity, insured status, and data on the primary diagnosis associated with the visit and secondary diagnoses that affect the course or cost of treatment. We used data from three states where housing status was available to achieve the broadest range of socio-economic and geographic diversity in the study. Only seven states -- 3 states included in our analysis plus Maryland, Georgia, Utah, and Wisconsin -- reported both housing status and a unique patient linkage number for both SID and SEDD in 2014. Housing status for Utah and Wisconsin's SID/SEDD was severely underreported, the hospital identifier was not available in Georgia's SID/SEDD, and there were very few (<10) pregnant women experiencing homelessness in Maryland's SID/SEDD; therefore, these states were not included in our analyses. The SEDD was used in combination with the SID to identify women experiencing homelessness, and the main analysis was performed using the SID.

© 2021 Yamamoto A et al. *JAMA Network Open.*

eTable 1. *International Classification of Diseases, Ninth Revision* (*ICD-9*) Diagnosis and Procedure Codes, Diagnosis-Related Group (DRG) Codes Used to Define Delivery Hospitalizations

**Inclusion Criteria**	**Codes**
ICD-9 Diagnosis	V27, 650
ICD-9 Procedure	72, 73.22, 73.59, 73.6, 74.0, 74.1, 74.2, 74.4, 74.99
DRG codes	765-768, 774-775
**Exclusion Criteria**	**Codes**
ICD-9 Diagnosis	630-639
ICD-9 Procedure	69.01, 69.51, 74.91, 75.0

© 2021 Yamamoto A et al. *JAMA Network Open.*

eTable 2. *International Classification of Diseases, Ninth Revision* (*ICD-9*) Diagnosis, and Procedure Codes Used to Define Delivery Outcomes

**Outcome/Covariate**	**ICD-9 Codes**
**Covariates (ICD-9 diagnosis)**	
Ever smoking	305.1, V15.82, 649.0
Previous cesarean delivery	654.2
Multiple births	V27.2 - V27.7, 651
**Obstetric complications during pregnancy (ICD-9 diagnosis)**	
Antepartum hemorrhage	641.1, 641.2, 641.3, 641.8, 641.9
Placental abnormalities (previa, abruptio, accreta)	641.0 - 641.2, 667
**Obstetric complications before, during and after labor (ICD-9 diagnosis)**	
Premature rupture of the membranes	658.10, 658.11, 658.13
Preterm labor	644.0, 644.2
Postpartum hemorrhage	666.0, 666.1, 666.2, 666.3
**Neonatal complications (ICD-9 diagnosis)**	
Fetal distress	656.3, 656.8, 659.7
Fetal growth restriction	656.5
Stillbirth	656.4, V27.1, V27.3, V27.4, V27.6, V27.7
**Delivery method (ICD-9 diagnosis and procedure)**	
Cesarean delivery	
ICD-9 diagnosis	699.7
ICD-9 procedure	74.0, 74.1, 74.2, 74.4, 74.99

© 2021 Yamamoto A et al. *JAMA Network Open.*

eTable 3. Comparison of Patient Outcomes Between Women Experiencing Homelessness vs Not Experiencing Homelessness

	**Adjusted Risk with Hospital Fixed Effects**		**Adjusted Risk Difference, comparing experiencing vs. not experiencing homelessness (95% CI)**
	**Experiencing homelessness (N=15,029)**	**Not experiencing homelessness (N=308,242)**	
**Obstetric complications during pregnancy**			
Antepartum hemorrhage	2.4% (1.1% to 3.7%)	1.6% (1.5% to 1.7%)	+0.8% (-0.6% to +2.2%)
Placental abnormalities	4.0% (2.4% to 5.5%)	2.0% (1.9% to 2.1%)	+1.9% (+0.4% to +3.5%)
**Obstetric complications before, during and after labor**			
Premature rupture of the membranes	6.2% (4.1% to 8.4%)	6.6% (6.4% to 6.7%)	-0.4% (-2.6% to +1.9%)
Preterm labor	10.5% (8.0% to 13.0%)	6.7% (6.5% to 6.9%)	+3.8% (+1.2% to +6.5%)
Postpartum hemorrhage	2.5% (0.4% to 4.5%)	3.6% (3.4% to 3.7%)	-1.1% (-3.2% to +1.0%)
**Neonatal complications**			
Fetal distress	18.8% (14.9% to 22.8%)	19.5% (19.2% to 19.8%)	-0.6% (-4.7% to +3.5%)
Fetal growth restriction	3.0% (1.4% to 4.7%)	2.7% (2.5% to 2.8%)	+0.4% (-1.4% to +2.1%)
Stillbirth	1.2% (0.3% to 2.1%)	0.8% (0.7% to 0.8%)	+0.5% (-0.5% to 1.4%)
**Delivery method**			
Cesarean delivery	36.9% (33.3% to 40.5%)	35.9% (35.6% to 36.1%)	+1.1% (-2.7% to +4.8%)
**Health services outcome**			
Cost	$6,306 ($6,054 to $6,559)	$5,888 ($5,864 to $5,913)	+$417 (+$156 to +$680)

Adjusted for patient characteristics (age, race/ethnicity, payer, ever smoker, multiple births, Elixhauser comorbidities). For the cesarean delivery outcome model, additionally adjusted for having had a previous cesarean delivery. Costs inflated to 2019 dollars using the Gross Domestic Product price index

© 2021 Yamamoto A et al. *JAMA Network Open.*

eTable 4. Patient Outcomes Excluding Comorbidities

	Adjusted Risk with Hospital Fixed Effects		Adjusted Risk Difference, comparing experiencing vs. not experiencing homelessness (95% CI)
	Experiencing homelessness (N=15,029)	Not experiencing homelessness (N=308,242)	
**Obstetric complications during pregnancy**			
Antepartum hemorrhage	2.9% (1.5% to 4.2%)	1.6% (1.5% to 1.7%)	+1.3% (-0.1% to +2.7%)
Placental abnormalities	4.4% (2.9% to 5.8%)	2.0% (1.9% to 2.1%)	+2.4% (+0.8% to +3.9%)
**Obstetric complications before, during and after labor**			
Premature rupture of the membranes	6.4% (4.3% to 8.5%)	6.6% (6.4% to 6.7%)	-0.2% (-2.4% to +2.0%)
Preterm labor	11.6% (9.0% to 14.1%)	6.6% (6.4% to 6.8%)	+5.0% (+2.3% to +7.6%)
Postpartum hemorrhage	2.6% (0.7% to 4.5%)	3.6% (3.4% to 3.7%)	-1.0% (-3.0% to +1.0%)
**Neonatal complications**			
Fetal distress	19.5% (15.7% to 23.3%)	19.4% (19.2% to 19.7%)	+0.1% (-3.9% to +4.1%)
Fetal growth restriction	3.1% (1.5% to 4.8%)	2.7% (2.5% to 2.8%)	+0.5% (-1.2% to +2.2%)
Stillbirth	1.2% (0.3% to 2.1%)	0.8% (0.7% to 0.8%)	+0.5% (-0.5% to +1.4%)
**Delivery method**			
Cesarean delivery	37.4% (33.8% to 40.9%)	35.8% (35.6% to 36.1%)	+1.5% (-2.2% to +5.2%)
**Health services outcome**			
Cost	$6,486 ($6,119 to $6,854)	$5,880 ($5,853 to $5,908)	+$606 (+$227 to +$985)

Adjusted for patient characteristics (age, race/ethnicity, payer, ever smoker, multiple births. For the cesarean delivery outcome model, additionally adjusted for having had a previous cesarean delivery. Costs inflated to 2019 dollars using the Gross Domestic Product price index.

© 2021 Yamamoto A et al. *JAMA Network Open.*

eTable 5. Patient Outcomes in Women Experiencing Homelessness vs Women Who Were Low Income and Housed

	**Adjusted Risk with Hospital Fixed Effects**		**Adjusted Risk Difference, comparing experiencing vs. not experiencing homelessness (95% CI)**
	**Experiencing homelessness (15,029)**	**Not experiencing homelessness (94,739)**	
**Obstetric complications during pregnancy**			
Antepartum hemorrhage	2.9% (1.6% to 4.2%)	1.5% (1.3% to 1.7%)	+1.4% (-0.1% to 2.9%)
Placental abnormalities	3.9% (2.3% to 5.4%)	1.9% (1.6% to 2.1%)	+2.0% (0.2% to 3.8%)
**Obstetric complications before, during and after labor**			
Premature rupture of the membranes	6.3% (4.5% to 8.1%)	6.5% (6.2% to 6.9%)	-0.2% (-2.3% to 1.8%)
Preterm labor	9.7% (7.5% to 11.9%)	6.7% (6.3% to 7.1%)	+3.0% (0.5% to 5.6%)
Postpartum hemorrhage	3.8% (2.2% to 5.4%)	3.6% (3.3% to 3.9%)	+0.2% (-1.6% to 2.1%)
**Neonatal complications**			
Fetal distress	19.9% (16.8% to 23.0%)	20.6% (20.0% to 21.2%)	-0.7% (-4.2% to 2.9%)
Fetal growth restriction	3.7% (2.3% to 5.1%)	2.4% (2.2% to 2.7%)	+1.3% (-0.3% to 2.9%)
Stillbirth	0.9% (0.0% to 1.8%)	0.8% (0.7% to 1.0%)	+0.1% (-0.9% to 1.1%)
**Delivery method**			
Cesarean delivery	38.2% (35.3% to 41.1%)	34.9% (34.4% to 35.5%)	+3.3% (-0.1% to 6.7%)
**Health services outcome**			
Cost	$6,226 ($5,971 to $6,480)	$5,712 ($5,669 to $5,756)	+$514 (+$231 to +$796)

Adjusted for patient characteristics (age, race/ethnicity, payer, ever smoker, multiple births, Elixhauser comorbidities). For the cesarean delivery outcome model, additionally adjusted for having had a previous cesarean delivery. Costs inflated to 2019 dollars using the Gross Domestic Product price index

© 2021 Yamamoto A et al. *JAMA Network Open.*

eTable 6. Patient Outcomes Without Adjusting for Hospital Fixed Effects (Comparing Across Hospitals)

	**Adjusted Risk No Hospital Fixed Effects**		**Adjusted Risk Difference, comparing experiencing vs. not experiencing homelessness (95% CI)**
	**Experiencing homelessness (N=15,029)**	**Not experiencing homelessness (N=308,242)**	
**Obstetric complications during pregnancy**			
Antepartum hemorrhage	1.6% (0.8% to 2.4%)	1.6% (1.6% to 1.7%)	-0.1% (-0.3% to +0.7%)
Placental abnormalities	2.5% (1.5% to 3.5%)	2.1% (2.0% to 2.2%)	+0.4% (-0.7% to +1.5%)
**Obstetric complications before, during and after labor**			
Premature rupture of the membranes	6.0% (4.5% to 7.5%)	6.6% (6.4% to 6.7%)	-0.6% (-2.1% to +0.9%)
Preterm labor	9.0% (7.1% to 10.9%)	6.7% (6.6% to 6.9%)	+2.2% (0.2% to +4.2%)
Postpartum hemorrhage	3.4% (2.3% to 4.5%)	3.5% (3.4% to 3.7%)	-0.1% (-1.3% to +1.0%)
**Neonatal complications**			
Fetal distress	23.4% (20.7% to 26.2%)	19.2% (19.0% to 19.5%)	+4.2% (+1.3% to +7.0%)
Fetal growth restriction	2.4% (1.5% to 3.2%)	2.7% (2.6% to 2.8%)	-0.3% (-1.2% to +0.6%)
Stillbirth	1.1% (0.4% to 1.7%)	0.8% (0.7% to 0.8%)	+0.3% (-0.4% to +1.0%)
**Delivery method**			
Cesarean delivery	31.8% (29.3% to 34.3%)	36.1% (35.9% to 36.4%)	-4.3% (-6.9% to -1.8%)
**Health services outcome**			
Cost	$5,925 ($5,672 to $6,179)	$5,901 ($5,874 to $5,929)	-$24 (-$238 to +$286)

Adjusted for patient characteristics (age, race/ethnicity, payer, ever smoker, multiple births, Elixhauser comorbidities). For the cesarean delivery outcome model, additionally adjusted for having had a previous cesarean delivery. Costs inflated to 2019 dollars using the Gross Domestic Product price index

© 2021 Yamamoto A et al. *JAMA Network Open.*

eTable 7. Delivery-Associated Costs by Different Modeling Approaches

	Adjusted Risk with Hospital FE		Adjusted Risk Difference, comparing experiencing vs. not experiencing homelessness (95% CI)
	**Experiencing homelessness (N=15,029)**	**Not experiencing homelessness (N=308,242)**	
GLM with log-link negative binomial distribution	$6,306<br>($6,054 to $6,559)	$5,888<br>($5,864 to $5,913)	+$417<br>(+$156 to +$680)
OLS	$6,471<br>($6,076 to $6,867)	$5,875<br>($5,846 to $5,905)	+$596<br>(+$182 to +$1,009)

Adjusted for patient characteristics (age, race/ethnicity, payer, ever smoker, multiple births, Elixhauser comorbidities). Costs inflated to 2019 dollars using the Gross Domestic Product price index

© 2021 Yamamoto A et al. *JAMA Network Open.*
